# Mycobacterium tuberculosis DprE1 Inhibitor OPC-167832 Is Active against Mycobacterium abscessus *In Vitro*

**DOI:** 10.1128/aac.01237-22

**Published:** 2022-11-09

**Authors:** Jickky Palmae Sarathy, Matthew D. Zimmerman, Martin Gengenbacher, Véronique Dartois, Thomas Dick

**Affiliations:** a Center for Discovery and Innovation, Hackensack Meridian Health, Nutley, New Jersey, USA; b Department of Medical Sciences, Hackensack Meridian School of Medicine, Nutley, New Jersey, USA; c Department of Microbiology and Immunology, Georgetown University, Washington, DC, USA

**Keywords:** OPC-167832, DprE1, SigA, *Mycobacterium abscessus*, NTM, nontuberculous mycobacteria

## Abstract

The antituberculosis candidate OPC-167832, an inhibitor of DprE1, was active against Mycobacterium abscessus. Resistance mapped to M. abscessus *dprE1*, suggesting target retention. OPC-167832 was bactericidal and did not antagonize activity of clinical anti-M. abscessus antibiotics. Due to its moderate potency compared to that against Mycobacterium tuberculosis, the compound lacked efficacy in a mouse model and is thus not a repurposing candidate. These results identify OPC-167832–DprE1 as a lead-target couple for a M. abscessus-specific optimization program.

## INTRODUCTION

The nontuberculous mycobacterium (NTM) and opportunistic pathogen Mycobacterium abscessus can establish extremely difficult to treat lung infections (1–3). Complex antibiotic regimens, typically consisting a macrolide (clarithromycin or azithromycin), the aminoglycoside amikacin, and a β-lactam (cefoxitin or imipenem), are administered often for years and show low cure rates (4–8). In brief, there is no reliable cure for M. abscessus lung disease. The M. abscessus drug pipeline is thinly populated (9), and new repurposing candidates and lead-target couples are sorely needed (10).

Decaprenylphosphoryl-β-d-ribose oxidase (DprE1) has emerged as an attractive target for antituberculosis (anti-TB) drug development (11–13). The enzyme, catalyzing the formation of decaprenyl-phospho-arabinose (DPA), is essential for growth and viability of Mycobacterium tuberculosis (14–16). DPA serves as a precursor for the synthesis of arabinogalactan, a critical component of the mycobacterial cell wall (14). Inhibitors of M. tuberculosis DprE1 have been identified from various structural scaffolds and show potent activity *in vitro* and in mouse models (13, 17, 18). BTZ-043, PBTZ-169, OPC-167832, and TBA-7371 have progressed to phase I or II clinical trials for TB (19).

Transposon mutagenesis studies have shown that M. abscessus *dprE1* (*mab_0192c*) is genetically essential (20). Whether M. abscessus DprE1 is a vulnerable target whose inhibition would translate into whole-cell antimicrobial activity has not been established. BTZ-043 and its analog PBTZ-169 have been tested for activity against M. abscessus, and both were found to be inactive (21, 22). This is likely due to an amino acid polymorphism in M. abscessus DprE1. BTZ-043 and PBTZ-169 form covalent adducts with cysteine 387 in M. tuberculosis DprE1 as their on-target mechanism of action (22–24). *M. abscesuss* DprE1 has alanine at the corresponding amino acid residue position, thus preventing covalent adduct formation and enzyme inhibition by the covalent inhibitors (22–24).

Here, we tested the growth inhibitory activity of the DprE1 inhibitors OPC-167832 and TBA-7371, which do not form covalent adducts with their target (25, 26). The MIC of the compounds against the type strain M. abscessus subsp. *abscessus* ATCC 19977 (American Type Culture Collection) was determined in Middlebrook 7H9 broth (BD) using the broth microdilution method with optical density at 600 nm (OD_600_) as the readout as described previously (27). The MIC was defined as 90% growth inhibition compared to the drug-free culture. While TBA-7371 (MedChem Express) was inactive (MIC > 100 μM), the dihydrocarbostyril OPC-167832 (MedChem Express) was found to be active (MIC = 5.2 μM). To determine whether the activity of OPC-167832 against the type strain was retained against the broader M. abscessus complex (28), MICs were measured against the reference strains of the two other subspecies, M. abscessus subsp. *bolletii* CCUG 50184^T^ and M. abscessus subsp. *massiliense* CCUG 48898^T^ (Culture Collection University of Goteborg), and against a panel of clinical isolates which include *erm41*-harboring macrolide-resistant strains (29, 30). Potency was largely consistent across the members of the M. abscessus complex, with MICs ranging from 5.2 to 15 μM (Table 1).

**TABLE 1:** MIC of OPC-167832 against M. abscessus complex

*M. abscessus* strain	*erm41* sequevar^*a*^	Clarithromycin susceptibility	MIC (μM) of^*b*^:	
			OPC-167832	Clarithromycin
Reference strains				
*M. abscessus* subsp. *abscessus* ATCC 19977	T28	Resistant	5.2	1.2
*M. abscessus* subsp. *bolletii* CCUG 50184-T	T28	Resistant	5.4	3.2
*M. abscessus* subsp. *massiliense* CCUG 48898-T	Deletion	Sensitive	6	0.6
Clinical isolates^*c*^				
*M. abscessus* subsp. *abscessus* Bamboo	C28	Sensitive	10	0.8
*M. abscessus* subsp. *abscessus* K21	C28	Sensitive	15	2
*M. abscessus* subsp. *abscessus* M9	T28	Resistant	10	5.4
*M. abscessus* subsp. *abscessus* M199	T28	Resistant	10	4.8
*M. abscessus* subsp. *abscessus* M337	T28	Resistant	7	2.9
*M. abscessus* subsp. *abscessus* M404	C28	Sensitive	8.5	0.8
*M. abscessus* subsp. *abscessus* M421	T28	Resistant	9.3	1.3
*M. abscessus* subsp. *bolletii* M232	T28	Resistant	8.8	5.4
*M. abscessus* subsp. *bolletii* M506	C28	Sensitive	6	0.6
*M. abscessus* subsp. *massiliense* M111	Deletion	Sensitive	11	0.7

a *erm41* is the methylase gene responsible for inducible clarithromycin resistance. The C28 and deletion sequevars are inactive *erm41* alleles and result in susceptibility to clarithromycin, while the T28 sequevar is functional and confers inducible resistance against clarithromycin (29, 30).

b MIC determination was carried out three times independently, and the results are presented as mean values. Clarithromycin (Sigma-Aldrich) was used as the assay control (34).

c *M. abscessus* Bamboo, M. abscessus K21, and the other clinical isolates were characterized and reported previously (32, 39, 40).

The micromolar concentration activity against the NTM is in stark contrast to the nanomolar concentration activities of OPC-167832 reported for M. tuberculosis (25). The dramatic *in vitro* potency difference of the TB drug candidate against M. abscessus suggests that OPC-167832 is likely not a repurposing candidate for the treatment of this lung disease. This was confirmed by *in vivo* pharmacokinetic-pharmacodynamic analyses. All experiments involving live animals were approved by the Institutional Animal Care and Use Committee of the Center for Discovery and Innovation, Hackensack Meridian Health (no. 269.030 and no. 265.015).

**FIG 1:**
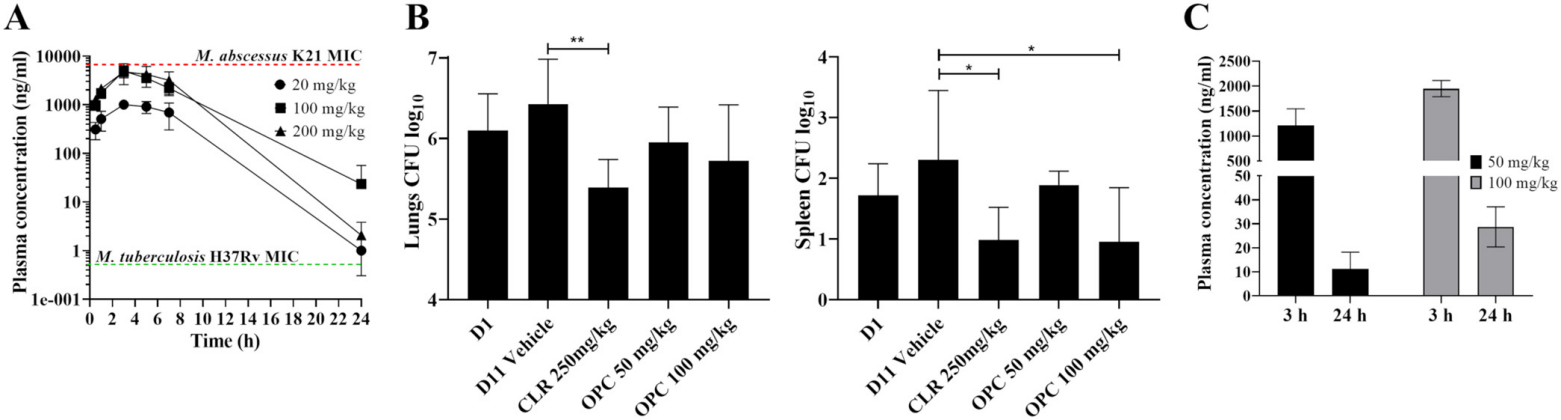
Pharmacokinetic profile and activity of OPC-167832 in mice. (A) Plasma concentration versus time profile of OPC-167832. Female CD-1 mice received a single dose of 20, 100, or 200 mg/kg of OPC-167832 formulated in 5% (wt/vol) gum arabic solution (Sigma-Aldrich) by oral gavage. Blood samples were collected from the tail vein at 0.5, 1, 3, 5, 7, and 24 h after drug administration, and the plasma concentration of OPC-167832 was measured by high-pressure liquid chromatography coupled to tandem mass spectrometry (LC-MS/MS). The MIC of OPC-167832 against M. abscessus K21 (15 μM [6,852.6 ng/mL]) (Table 1) is indicated by the red dotted line. The reported MIC of OPC-167832 against M. tuberculosis H37Rv (1.1 nM [0.5 ng/mL]) is indicated by the green dotted line (25). (B) *In vivo* efficacy of OPC-167832 against M. abscessus in a NOD SCID mouse model. NOD SCID mice were infected intranasally with M. abscessus K21. Starting 1 day postinfection (D1), OPC-167832 (50 or 100 mg/kg, formulated in 5% gum arabic solution), the positive control, clarithromycin (250 mg/kg, formulated in 0.5% carboxymethyl cellulose–0.5% Tween 80–sterile water), or drug-free OPC-167832 vehicle was orally administered to infected mice for 10 consecutive days via oral gavage. Twenty-four hours after the last dose (11 days postinfection), all mice were euthanized, and their lungs and spleen were aseptically removed prior to homogenization. Serial dilutions of organ homogenates were plated onto Middlebrook 7H11 agar (BD) to quantify lung and spleen bacterial load on day 1 postinfection and after administration of drug-free vehicle (D11 Vehicle), OPC-167832 (OPC), and clarithromycin (CLR). The mean and standard deviation are shown for each treatment group (*n* = 6). Statistical significance was determined using one-way analysis of variance (ANOVA) for multiple comparisons and Dunnett’s posttest (*, *P* < 0.05; **, *P* < 0.01). The experiment was carried out twice, showing similar results, and one representative data set is shown. (C) Plasma concentrations of OPC-167832 in infected NOD SCID mice 3 and 24 h after the last dose in the efficacy experiment shown in panel B. The graphs were generated using GraphPad Prism 9 software.

The plasma concentration-time profile upon oral administration of OPC-167832 in uninfected CD-1 mice (Charles River Laboratories) was determined by measuring the plasma concentrations of the compound via high-pressure liquid chromatography coupled to tandem mass spectrometry (LC-MS/MS) as described previously (31). Dosing at 20 or 100 mg/kg of body weight resulted in a plasma concentration versus time curve above the MIC for M. tuberculosis (25) for most of a 24-h interval; however, the MIC for M. abscessus was not reached (Fig. 1A). As increasing the dose from 100 to 200 mg/kg did not result in a significant increase of exposure (Fig. 1A), 100 mg/kg was chosen as the highest dose for an efficacy study in a M. abscessus mouse model. NOD.CB17-Prkdc^scid^/NCrCrl mice (NOD SCID; Charles River Laboratories) were infected with M. abscessus K21 as described previously (32) and treated once daily for 10 days with orally administered OPC-167832 (50 or 100 mg/kg), clarithromycin (250 mg/kg) as the positive control, or drug-free vehicle. As expected, OPC-167832 treatment did not result in a statistically significant reduction of the lung bacterial burden (Fig. 1B). Plasma concentrations of OPC-167832 were measured 3 h and 24 h after the last dose, confirming similar concentrations in infected and naive mice (Fig. 1A and C). Together, these *in vivo* analyses suggest that OPC-167832 is not a repurposing candidate for M. abscessus lung disease due to its moderate micromolar concentration *in vitro* potency compared to its nanomolar concentration activity against M. tuberculosis. It is interesting to note that OPC-167832 at 100 mg/kg showed a weak effect on the bacterial burden in the spleen, similar to the positive control, clarithromycin, at 250 mg/kg (Fig. 1B). The reason for this apparent organ-specific effect remains to be determined and may involve differential drug penetration and/or differences in the pathophysiology of the bacteria.

To determine whether OPC-167832 retains DprE1 as its target in M. abscessus and inform future lead optimization efforts, spontaneously resistant mutants were isolated using the type strain M. abscessus ATCC 19977 on Middlebrook 7H10 agar as described previously (31). A total of 20 × 10^9^ CFU were plated on agar medium containing 16× MIC (MIC = 5.2 μM) (Table 1), the lowest OPC-167832 concentration that suppressed growth of wild-type colonies, resulting in a frequency of resistance of 10^−9^/CFU. The experiment was repeated with another independently grown culture, yielding a similar frequency of resistance. Twelve randomly selected OPC-167832-resistant strains (OPC_RM1 to OPC_RM12) from the two selection experiments achieved pronounced resistance with an ~30- to 100-fold-higher MIC than the parent strain (Table 2). Whole-genome sequencing (Novogene Corporation, Inc.), followed by Sanger sequencing (Genewiz, Inc.), revealed that the 12 OPC-167832-resistant strains comprised two genotypic classes. Six strains (OPC_RM1 to OPC_RM6) harbored four different missense mutations in the M. abscessus homolog of *dprE1*, while the other six resistant strains (OPC_RM7 to OPC_RM12) harbored five different missense mutations in the homolog of *sigA* (*mab_3009*), which encodes the essential sigma factor A that assists the RNA polymerase in recognizing promoters of target genes (Table 2) (33).

**TABLE 2:** Characterization of spontaneous and engineered OPC-167832-resistant M. abscessus ATCC 19977 strains

Strain	MIC (μM) of^*a*^:		Candidate resistance gene	Polymorphisms in candidate resistance gene^*b*^	Polymorphisms in other genes^*c*^
	OPC-167832	Clarithromycin			
Wild type (WT)	5.2	1.2	NA^*d*^	NA	NA
Spontaneous mutants (culture batch)^*e*^					
OPC_RM1 (1)	>500	1.5	*dprE1*	G745A/Gly249Arg	None
OPC_RM2 (1)	230	1.6	*dprE1*	G103A/Ala35Thr	*mab_0020* A104C/Gly249Arg
OPC_RM3 (1)	220	1.8	*dprE1*	T872G/Val291Gly	None
OPC_RM4 (2)	>500	1.6	*dprE1*	G745A/Gly249Arg	None
OPC_RM5 (2)	180	1.2	*dprE1*	C484T/Pro162Ser	None
OPC_RM6 (2)	380	1.2	*dprE1*	G103A/Ala35Thr	*mab_0341* C38T/Ala13Val
OPC_RM7 (1)	>500	2	*sigA*	G787A/Gly263Ser	*mab_1186c* Δ29G
OPC_RM8 (1)	>500	2.3	*sigA*	C822A/Phe274Leu	*mab_2152* Δ−14G
OPC_RM9 (2)	>500	1.8	*sigA*	A509G/Tyr170Cys	*mab_1612* G901A/Glu301Lys
OPC_RM10 (2)	>500	1.6	*sigA*	T589G/Tyr197Asp	None
OPC_RM11 (2)	>500	1.8	*sigA*	G787A/Gly263Ser	*mab_4225c* Δ167CT
OPC_RM12 (2)	>500	2	*sigA*	G895A/Ala299Thr	*mab_0938c* Δ752T; *mab_0389c* A223C/Lys75Glu
Engineered strains^*f*^					
WT/pMV262 empty	6	1.8	NA	NA	ND^*g*^
WT/pMV262 dprE1*	>500	1.8	NA	NA	ND
WT/pMV262 *dprE1*	27	1.5	NA	NA	ND
OPC_RM7/pMV262 empty	>500	1.8	*sigA*	G787A/Gly263Ser	*mab_1186c*: Δ29G
OPC_RM7/pMV262 *sigA*	80	1.4	*sigA*	G787A/Gly263Ser	*mab_1186c*: Δ29G

a MIC determination was carried out three times independently and the results are presented as mean values. Clarithromycin was used as the assay control.

b The spontaneously resistant strains OPC_RM1 to OPC_RM12 were subjected to whole-genome sequencing, followed by confirmation by Sanger sequencing. The primers used for Sanger sequencing are described in Table S1. The identified mutations in the resistant strains are detailed by the changes in the DNA/amino acid sequence of the affected genes and proteins, respectively.

c Non-*dprE1* and non-*sigA* polymorphisms detected by whole-genome sequencing. Consistent polymorphism in other genes were not observed. The identified mutations in the resistant strains are detailed by the changes in the DNA/amino acid sequence of the affected genes and proteins, respectively.

d NA, not applicable.

e Twelve spontaneous OPC-167832-resistant strains (OPC_RM1 to OPC_RM12), isolated from two independent culture batches, were randomly selected for characterization.

f WT/pMV262 empty, wild-type M. abscessus ATCC 19977 strain harboring the pMV262-*hsp60* expression system without any inserted genes; WT/pMV262 dprE1*, wild-type strain expressing mutant *dprE1* (from the spontaneous mutant strain OPC_RM1) carried by pMV262 under the control of *hsp60* promoter; WT/pMV262 *dprE1*, wild-type strain expressing wild-type *dprE1* carried by pMV262 under the control of *hsp60* promoter; OPC_RM7/pMV262 empty, OPC-167832 resistant strain OPC_RM7 (possessing mutant *sigA*) harboring the pMV262-*hsp60* expression system without any inserted genes; OPC_RM7/pMV262 *sigA*, resistant strain OPC_RM7 expressing wild-type *sigA* carried by pMV262 under the control of *hsp60* promoter.

g ND, not determined.

To confirm that the observed polymorphisms detected in *dprE1* and *sigA* are indeed responsible for resistance to OPC-167832, merodiploid strains were engineered using custom-synthesized pMV262-*hsp60*-based expression systems (Genewiz, Inc.) as described previously (34). To confirm involvement of *dprE1* missense mutations, a copy of the mutant *dprE1* allele from a representative resistant strain (OPC_RM1) (Table 2) was constitutively expressed under the control of the *hsp60* promoter in wild-type M. abscessus ATCC 19977. As expected, the strain expressing the mutant *dprE1* allele displayed high-level resistance to OPC-167832 (Table 2). To exclude the possibility that the observed resistance was caused by mere overexpression of the *dprE1* gene, as opposed to the missense mutation harbored by the mutant *dprE1* allele, the wild-type allele of *dprE1* was expressed under the control of the *hsp60* promoter in wild-type M. abscessus ATCC 19977. This resulted in low-level resistance to OPC-167832 (Table 2), indicating that the missense mutations are the major contributors to the resistance phenotype. Together, these genetic analyses suggest that DprE1 is a target of OPC-167832 in M. abscessus. To confirm that the observed polymorphisms in *sigA* cause resistance to OPC-167832, one representative *sigA* mutant strain (OPC_RM7) (Table 2) was complemented with a wild-type copy of the *sigA* gene that was constitutively expressed under the control of the *hsp60* promoter. Expression of wild-type *sigA* in the mutant background partially restored sensitivity to OPC-167832 (Table 2), suggesting that the missense mutations observed in *sigA* are the cause of resistance to OPC-167832. It is interesting to note that *sigA* mutations have not been reported to be involved in resistance of M. tuberculosis to DprE1 inhibitors. However, a few reports describe mutations in *sigA* causing drug resistance in other bacteria, apparently by reprogramming the transcriptome (35, 36). How mutations in M. abscessus *sigA* cause resistance against OPC-167832 remains to be determined.

**FIG 2:**
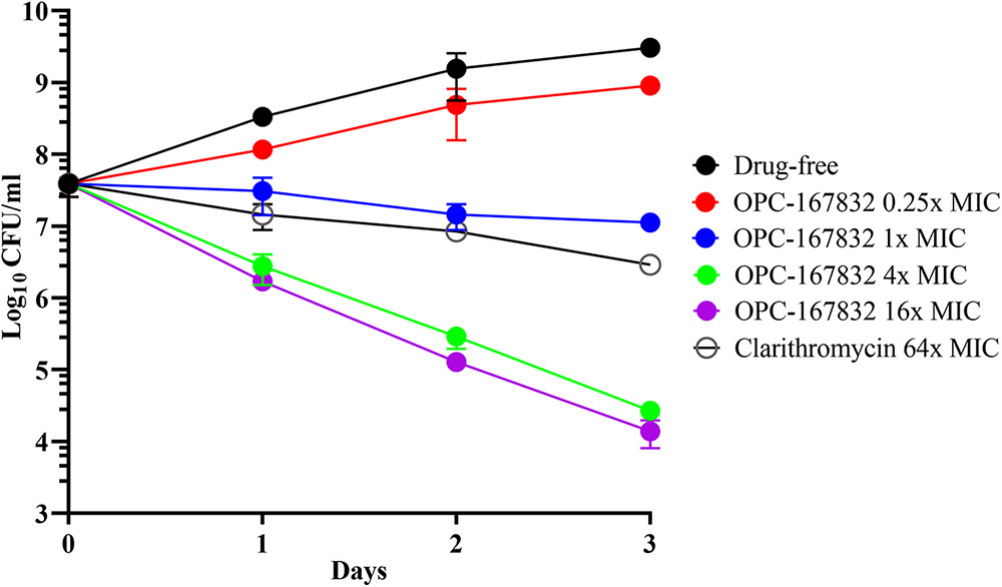
*In vitro* time-kill activity of OPC-167832 against M. abscessus ATCC 19977. M. abscessus cultures were grown in Middlebrook 7H9 and treated with 0.25×, 1×, 4×, and 16× MIC of OPC-167832 (MIC = 5.2 μM) (Table 1) over a period of 3 days, and CFU per milliliter were measured by plating samples on Middlebrook 7H10 agar. Clarithromycin was used as a negative control at 64× MIC (MIC = 1.2 μM) (Table 1). The experiment was carried out three times independently, and the results are presented as mean values with standard deviations displayed as error bars. The graph was generated using GraphPad Prism 9 software.

To further evaluate the attractiveness of OPC-167832–DprE1 as a lead-target couple, *in vitro* bactericidal activity and *in vitro* drug-drug potency interactions with anti-M. abscessus antibiotics were determined using M. abscessus ATCC 19977 as described previously (27). OPC-167832 was bactericidal, with a 3-log kill at 4× MIC (Fig. 2). The absence of antagonism with clarithromycin, amikacin (Sigma-Aldrich), cefoxitin (MedChem Express), or imipenem (Cayman Chemical) (Table 3), together with the clean drug-drug interaction profile of OPC-167832 as required under multidrug TB therapy (25, 37, 38), suggests that dihydrocarbostyril analogs are compatible with the current standard of care for M. abscessus lung disease.

**TABLE 3:** *In vitro* drug-drug potency interaction between OPC-167832 and selected clinically used drugs against M. abscessus ATCC 19977

Drug^*a*^	Class	Target	MIC (μM)^*b*^		FICI^*c*^	Outcome^*c*^
			Alone	In combination		
OPC-167832	3,4-Dihydrocarbostyril	DprE1	5.2	1.9	0.6	Additivity
Clarithromycin	Macrolide	50S ribosomal subunit	1.2	0.3		

OPC-167832	3,4-Dihydrocarbostyril	DprE1	5.2	1.6	0.9	Additivity
Amikacin	Aminoglycoside	30S ribosomal subunit	30	17		

OPC-167832	3,4-Dihydrocarbostyril	DprE1	5.2	1.7	0.6	Additivity
Cefoxitin	β-Lactam	Peptidoglycan biosynthesis transpeptidases	26	7.8		

OPC-167832	3,4-Dihydrocarbostyril	DprE1	5.2	1.5	0.4	Synergy
Imipenem	β-Lactam	Peptidoglycan biosynthesis transpeptidases	25	3.6		

a To determine possible antagonisms between OPC-167832 and clinically used drugs, checkerboard analyses were carried using a 96-well plate format (41, 42). The effect of serially diluted OPC-167832 ranging from 0.39 μM to 25 μM was tested against the partner drugs ranging from 0.49 μM to 250 μM.

b MIC determination was carried out three times independently, and the results are presented as mean values.

c The fractional inhibitory concentration index (FICI) was calculated as [(MIC of partner drug in combination/MIC of partner drug alone) + (MIC of OPC-167832 in combination/MIC of OPC-167832 alone)]. An FICI value of ≤0.5 indicates synergy, an FICI value of 0.5 to 4 indicates additivity (no interaction), and an FICI value of >4 indicates antagonism (43).

In conclusion, we identified OPC-167832 as the first whole-cell active inhibitor of M. abscessus DprE1, thus validating DprE1 as a vulnerable target in the opportunistic pathogen. The 1,000-fold-weaker activity of OPC-167832 against M. abscessus compared to M. tuberculosis results in unfavorable pharmacokinetic-pharmacodynamic parameters and lack of efficacy in a mouse model of M. abscessus infection. Thus, the TB drug candidate is unlikely to present a repurposing candidate for the treatment of M. abscessus lung disease. The reason for the pronounced potency difference against the two mycobacterial species remains to be determined and may involve target binding, uptake/excretion, or intrabacterial metabolism (10). If the basis for the differential potency can be elucidated, OPC-167832 may present an attractive chemical starting point for a rational, pathogen-specific lead optimization program.

## References

[1] Tortoli E. 2019. The taxonomy of the genus Mycobacterium, p 1–10. *In* Velayati AA, Farnia P (ed), Nontuberculous mycobacteria (NTM): microbiological, clinical and geographical distribution. Academic Press, London, United Kingdom.

[2] Jarand J, Levin A, Zhang L, Huitt G, Mitchell JD, Daley CL. 2011. Clinical and microbiologic outcomes in patients receiving treatment for *Mycobacterium abscessus* pulmonary disease. Clin Infect Dis 52:565–571. 10.1093/cid/ciq237. 21292659

[3] Daniel-Wayman S, Abate G, Barber DL, Bermudez LE, Coler RN, Cynamon MH, Daley CL, Davidson RM, Dick T, Floto RA, Henkle E, Holland SM, Jackson M, Lee RE, Nuermberger EL, Olivier KN, Ordway DJ, Prevots DR, Sacchettini JC, Salfinger M, Sassetti CM, Sizemore CF, Winthrop KL, Zelazny AM. 2019. Advancing translational science for pulmonary nontuberculous mycobacterial infections. A road map for research. Am J Respir Crit Care Med 199:947–951. 10.1164/rccm.201807-1273PP. 30428263 PMC6467310

[4] Daley CL, Iaccarino JM, Lange C, Cambau E, Wallace RJ, Andrejak C, Böttger EC, Brozek J, Griffith DE, Guglielmetti L, Huitt GA, Knight SL, Leitman P, Marras TK, Olivier KN, Santin M, Stout JE, Tortoli E, van Ingen J, Wagner D, Winthrop KL. 2020. Treatment of nontuberculous mycobacterial pulmonary disease: an official ATS/ERS/ESCMID/IDSA clinical practice guideline. Clin Infect Dis 71:e1–e36. 10.1093/cid/ciaa241. 32628747 PMC7768748

[5] Haworth CS, Banks J, Capstick T, Fisher AJ, Gorsuch T, Laurenson IF, Leitch A, Loebinger MR, Milburn HJ, Nightingale M, Ormerod P, Shingadia D, Smith D, Whitehead N, Wilson R, Floto RA. 2017. British Thoracic Society guidelines for the management of non-tuberculous mycobacterial pulmonary disease (NTM-PD). Thorax 72:ii1–ii64. 10.1136/thoraxjnl-2017-210927. 29054853

[6] Chen J, Zhao L, Mao Y, Ye M, Guo Q, Zhang Y, Xu L, Zhang Z, Li B, Chu H. 2019. Clinical efficacy and adverse effects of antibiotics used to treat *Mycobacterium abscessus* pulmonary disease. Front Microbiol 10:1977. 10.3389/fmicb.2019.01977. 31507579 PMC6716072

[7] Kwak N, Dalcolmo MP, Daley CL, Eather G, Gayoso R, Hasegawa N, Jhun BW, Koh W-J, Namkoong H, Park J, Thomson R, van Ingen J, Zweijpfenning SM, Yim J-J. 2019. *Mycobacterium abscessus* pulmonary disease: individual patient data meta-analysis. Eur Respir J 54:1801991. 10.1183/13993003.01991-2018. 30880280

[8] Koh W-J, Jeong B-H, Kim S-Y, Jeon K, Park KU, Jhun BW, Lee H, Park HY, Kim DH, Huh HJ, Ki C-S, Lee NY, Kim HK, Choi YS, Kim J, Lee S-H, Kim CK, Shin SJ, Daley CL, Kim H, Kwon OJ. 2017. Mycobacterial characteristics and treatment outcomes in *Mycobacterium abscessus* lung disease. Clin Infect Dis 64:309–316. 10.1093/cid/ciw724. 28011608

[9] Egorova A, Jackson M, Gavrilyuk V, Makarov V. 2021. Pipeline of anti-*Mycobacterium abscessus* small molecules: repurposable drugs and promising novel chemical entities. Med Res Rev 41:2350–2387. 10.1002/med.21798. 33645845 PMC8217127

[10] Wu M-L, Aziz DB, Dartois V, Dick T. 2018. NTM drug discovery: status, gaps and the way forward. Drug Discov Today 23:1502–1519. 10.1016/j.drudis.2018.04.001. 29635026 PMC6078814

[11] Christophe T, Jackson M, Jeon HK, Fenistein D, Contreras-Dominguez M, Kim J, Genovesio A, Carralot J-P, Ewann F, Kim EH, Lee SY, Kang S, Seo MJ, Park EJ, Skovierová H, Pham H, Riccardi G, Nam JY, Marsollier L, Kempf M, Joly-Guillou M-L, Oh T, Shin WK, No Z, Nehrbass U, Brosch R, Cole ST, Brodin P. 2009. High content screening identifies decaprenyl-phosphoribose 2′ epimerase as a target for intracellular antimycobacterial inhibitors. PLoS Pathog 5:e1000645. 10.1371/journal.ppat.1000645. 19876393 PMC2763345

[12] Makarov V, Manina G, Mikusova K, Möllmann U, Ryabova O, Saint-Joanis B, Dhar N, Pasca MR, Buroni S, Lucarelli AP, Milano A, De Rossi E, Belanova M, Bobovska A, Dianiskova P, Kordulakova J, Sala C, Fullam E, Schneider P, McKinney JD, Brodin P, Christophe T, Waddell S, Butcher P, Albrethsen J, Rosenkrands I, Brosch R, Nandi V, Bharath S, Gaonkar S, Shandil RK, Balasubramanian V, Balganesh T, Tyagi S, Grosset J, Riccardi G, Cole ST. 2009. Benzothiazinones kill *Mycobacterium tuberculosis* by blocking arabinan synthesis. Science 324:801–804. 10.1126/science.1171583. 19299584 PMC3128490

[13] Chikhale RV, Barmade MA, Murumkar PR, Yadav MR. 2018. Overview of the development of DprE1 inhibitors for combating the menace of tuberculosis. J Med Chem 61:8563–8593. 10.1021/acs.jmedchem.8b00281. 29851474

[14] Wolucka BA. 2008. Biosynthesis of d-arabinose in mycobacteria—a novel bacterial pathway with implications for antimycobacterial therapy. FEBS J 275:2691–2711. 10.1111/j.1742-4658.2008.06395.x. 18422659

[15] Kolly GS, Boldrin F, Sala C, Dhar N, Hartkoorn RC, Ventura M, Serafini A, McKinney JD, Manganelli R, Cole ST. 2014. Assessing the essentiality of the decaprenyl-phospho-d-arabinofuranose pathway in *Mycobacterium tuberculosis* using conditional mutants. Mol Microbiol 92:194–211. 10.1111/mmi.12546. 24517327

[16] Mikusová K, Huang H, Yagi T, Holsters M, Vereecke D, D'Haeze W, Scherman MS, Brennan PJ, McNeil MR, Crick DC. 2005. Decaprenylphosphoryl arabinofuranose, the donor of the d-arabinofuranosyl residues of mycobacterial arabinan, is formed via a two-step epimerization of decaprenylphosphoryl ribose. J Bacteriol 187:8020–8025. 10.1128/JB.187.23.8020-8025.2005. 16291675 PMC1291263

[17] Piton J, Foo CS-Y, Cole ST. 2017. Structural studies of *Mycobacterium tuberculosis* DprE1 interacting with its inhibitors. Drug Discov Today 22:526–533. 10.1016/j.drudis.2016.09.014. 27666194

[18] Gawad J, Bonde C. 2018. Decaprenyl-phosphoryl-ribose 2′-epimerase (DprE1): challenging target for antitubercular drug discovery. Chem Cent J 12:72. 10.1186/s13065-018-0441-2. 29936616 PMC6015584

[19] World Health Organization. 2021. 2020 antibacterial agents in clinical and preclinical development: an overview and analysis. https://www.who.int/publications/i/item/9789240021303.

[20] Rifat D, Chen L, Kreiswirth BN, Nuermberger EL. 2021. Genome-wide essentiality analysis of *Mycobacterium abscessus* by saturated transposon mutagenesis and deep sequencing. mBio 12:e01049-21. 10.1128/mBio.01049-21. 34126767 PMC8262987

[21] Makarov V, Lechartier B, Zhang M, Neres J, van der Sar AM, Raadsen SA, Hartkoorn RC, Ryabova OB, Vocat A, Decosterd LA, Widmer N, Buclin T, Bitter W, Andries K, Pojer F, Dyson PJ, Cole ST. 2014. Towards a new combination therapy for tuberculosis with next generation benzothiazinones. EMBO Mol Med 6:372–383. 10.1002/emmm.201303575. 24500695 PMC3958311

[22] Shi J, Lu J, Wen S, Zong Z, Huo F, Luo J, Liang Q, Li Y, Huang H, Pang Y. 2018. *In vitro* activity of PBTZ169 against multiple *Mycobacterium* species. Antimicrob Agents Chemother 62:e01314-18. 10.1128/AAC.01314-18. 30150479 PMC6201125

[23] Batt SM, Jabeen T, Bhowruth V, Quill L, Lund PA, Eggeling L, Alderwick LJ, Fütterer K, Besra GS. 2012. Structural basis of inhibition of *Mycobacterium tuberculosis* DprE1 by benzothiazinone inhibitors. Proc Natl Acad Sci USA 109:11354–11359. 10.1073/pnas.1205735109. 22733761 PMC3396498

[24] Landge S, Mullick AB, Nagalapur K, Neres J, Subbulakshmi V, Murugan K, Ghosh A, Sadler C, Fellows MD, Humnabadkar V, Mahadevaswamy J, Vachaspati P, Sharma S, Kaur P, Mallya M, Rudrapatna S, Awasthy D, Sambandamurthy VK, Pojer F, Cole ST, Balganesh TS, Ugarkar BG, Balasubramanian V, Bandodkar BS, Panda M, Ramachandran V. 2015. Discovery of benzothiazoles as antimycobacterial agents: synthesis, structure-activity relationships and binding studies with *Mycobacterium tuberculosis* decaprenylphosphoryl-β-d-ribose 2′-oxidase. Bioorg Med Chem 23:7694–7710. 10.1016/j.bmc.2015.11.017. 26643218

[25] Hariguchi N, Chen X, Hayashi Y, Kawano Y, Fujiwara M, Matsuba M, Shimizu H, Ohba Y, Nakamura I, Kitamoto R, Shinohara T, Uematsu Y, Ishikawa S, Itotani M, Haraguchi Y, Takemura I, Matsumoto M. 2020. OPC-167832, a novel carbostyril derivative with potent antituberculosis activity as a DprE1 inhibitor. Antimicrob Agents Chemother 64:e02020-19. 10.1128/AAC.02020-19. 32229496 PMC7269503

[26] Shirude PS, Shandil R, Sadler C, Naik M, Hosagrahara V, Hameed S, Shinde V, Bathula C, Humnabadkar V, Kumar N, Reddy J, Panduga V, Sharma S, Ambady A, Hegde N, Whiteaker J, McLaughlin RE, Gardner H, Madhavapeddi P, Ramachandran V, Kaur P, Narayan A, Guptha S, Awasthy D, Narayan C, Mahadevaswamy J, Vishwas KG, Ahuja V, Srivastava A, Prabhakar KR, Bharath S, Kale R, Ramaiah M, Choudhury NR, Sambandamurthy VK, Solapure S, Iyer PS, Narayanan S, Chatterji M. 2013. Azaindoles: noncovalent DprE1 inhibitors from scaffold morphing efforts, kill *Mycobacterium tuberculosis* and are efficacious *in vivo*. J Med Chem 56:9701–9708. 10.1021/jm401382v. 24215368

[27] Sarathy JP, Ganapathy US, Zimmerman MD, Dartois V, Gengenbacher M, Dick T. 2020. TBAJ-876, a 3,5-dialkoxypyridine analogue of bedaquiline, is active against *Mycobacterium abscessus*. Antimicrob Agents Chemother 64:e02404-19. 10.1128/AAC.02404-19. 31964791 PMC7179298

[28] Adekambi T, Sassi M, van Ingen J, Drancourt M. 2017. Reinstating *Mycobacterium massiliense* and *Mycobacterium bolletii* as species of the *Mycobacterium abscessus* complex. Int J Syst Evol Microbiol 67:2726–2730. 10.1099/ijsem.0.002011. 28820087

[29] Nash KA, Brown-Elliott BA, Wallace RJ. 2009. A novel gene, *erm*(41), confers inducible macrolide resistance to clinical isolates of *Mycobacterium abscessus* but is absent from *Mycobacterium chelonae*. Antimicrob Agents Chemother 53:1367–1376. 10.1128/AAC.01275-08. 19171799 PMC2663066

[30] Bastian S, Veziris N, Roux A-L, Brossier F, Gaillard J-L, Jarlier V, Cambau E. 2011. Assessment of clarithromycin susceptibility in strains belonging to the *Mycobacterium abscessus* group by *erm*(41) and *rrl* sequencing. Antimicrob Agents Chemother 55:775–781. 10.1128/AAC.00861-10. 21135185 PMC3028756

[31] Ganapathy US, González del Rio R, Cacho-Izquierdo M, Ortega F, Lelièvre J, Barros-Aguirre D, Lindman M, Dartois V, Gengenbacher M, Dick T. 2021. A leucyl-tRNA synthetase inhibitor with broad-spectrum antimycobacterial activity. Antimicrob Agents Chemother 65:e02420-20. 10.1128/AAC.02420-20. 33558292 PMC8092876

[32] Dick T, Shin SJ, Koh W-J, Dartois V, Gengenbacher M. 2020. Rifabutin is active against *Mycobacterium abscessus* in mice. Antimicrob Agents Chemother 64:e01943-19. 10.1128/AAC.01943-19. 31767722 PMC6985736

[33] Gomez M, Doukhan L, Nair G, Smith I. 1998. *sigA* is an essential gene in *Mycobacterium smegmatis*. Mol Microbiol 29:617–628. 10.1046/j.1365-2958.1998.00960.x. 9720877

[34] Negatu DA, Beuchel A, Madani A, Alvarez N, Chen C, Aragaw WW, Zimmerman MD, Laleu B, Gengenbacher M, Dartois V, Imming P, Dick T. 2021. Piperidine-4-carboxamides target DNA gyrase in *Mycobacterium abscessus*. Antimicrob Agents Chemother 65:e00676-21. 10.1128/AAC.00676-21. 34001512 PMC8284461

[35] Burian J, Yim G, Hsing M, Axerio-Cilies P, Cherkasov A, Spiegelman GB, Thompson CJ. 2013. The mycobacterial antibiotic resistance determinant WhiB7 acts as a transcriptional activator by binding the primary sigma factor SigA (RpoV). Nucleic Acids Res 41:10062–10076. 10.1093/nar/gkt751. 23990327 PMC3905903

[36] Lee YH, Helmann JD. 2014. Mutations in the primary sigma factor σ^A^ and termination factor Rho that reduce susceptibility to cell wall antibiotics. J Bacteriol 196:3700–3711. 10.1128/JB.02022-14. 25112476 PMC4248801

[37] Tasneen R, Garcia A, Converse PJ, Zimmerman MD, Dartois V, Kurbatova E, Vernon AA, Carr W, Stout JE, Dooley KE, Nuermberger EL. 2022. Novel regimens of bedaquiline-pyrazinamide combined with moxifloxacin, rifabutin, delamanid and/or OPC-167832 in murine tuberculosis models. Antimicrob Agents Chemother 66:e02398-21. 10.1128/aac.02398-21. 35315690 PMC9017355

[38] Robertson GT, Ramey ME, Massoudi LM, Carter CL, Zimmerman M, Kaya F, Graham BG, Gruppo V, Hastings C, Woolhiser LK, Scott DWL, Asay BC, Eshun-Wilson F, Maidj E, Podell BK, Vásquez JJ, Lyons MA, Dartois V, Lenaerts AJ. 2021. Comparative analysis of pharmacodynamics in the C3HeB/FeJ mouse tuberculosis model for DprE1 inhibitors TBA-7371, PBTZ169 and OPC-167832. Antimicrob Agents Chemother 65:e00583-21. 10.1128/AAC.00583-21. 34370580 PMC8522729

[39] Yee M, Klinzing D, Wei J-R, Gengenbacher M, Rubin EJ, Dick T. 2017. Draft genome sequence of *Mycobacterium abscessus* Bamboo. Genome Announc 5:e00388-17. 10.1128/genomeA.00388-17. 28522728 PMC5477336

[40] Aziz DB, Low JL, Wu M-L, Gengenbacher M, Teo JW, Dartois V, Dick T. 2017. Rifabutin is active against *Mycobacterium abscessus* complex. Antimicrob Agents Chemother 61:e00155-17. 10.1128/AAC.00155-17. 28396540 PMC5444174

[41] Hsieh MH, Yu CM, Yu VL, Chow JW. 1993. Synergy assessed by checkerboard. A critical analysis. Diagn Microbiol Infect Dis 16:343–349. 10.1016/0732-8893(93)90087-N. 8495592

[42] Kaushik A, Makkar N, Pandey P, Parrish N, Singh U, Lamichhane G. 2015. Carbapenems and rifampin exhibit synergy against *Mycobacterium tuberculosis* and *Mycobacterium abscessus*. Antimicrob Agents Chemother 59:6561–6567. 10.1128/AAC.01158-15. 26259792 PMC4576034

[43] Odds FC. 2003. Synergy, antagonism, and what the chequerboard puts between them. J Antimicrob Chemother 52:1. 10.1093/jac/dkg301. 12805255

