# Supplementary material for: Mycobacterium tuberculosis DprE1 Inhibitor OPC-167832 Is Active against Mycobacterium abscessus In Vitro
Source: Antimicrob Agents Chemother. 2022 Nov 9;66(12):e01237-22. doi: 10.1128/aac.01237-22 (PMC9765218; doi:10.1128/aac.01237-22)
Supplement: Supplemental file 1 — Table S1. Download aac.01237-22-s0001.pdf, PDF file, 0.1 MB [file aac.01237-22-s0001.pdf]

TABLE S1 Primers used for Sanger sequencing of *M. abscessus dprE1* and *sigA* in the spontaneous OPC-167832-resistant strains[a]

Primer name	Sequence (5’ → 3’)	Purpose[b]
dprE1_1_Fwd	GACTTCGGCCTGTATGTGCTGTTCCTGCAC	PCR amplification and sequencing of *dprE1* + ~200 bp upstream start codon
dprE1_1_Rev	TTGCACGCGTGGTCGCCGTAGGAG	Sequencing of *dprE1*
dprE1_2_Fwd	CGTCGACGTCATTGCCGAGG	Sequencing of *dprE1*
dprE1_2_Rev	TGCGAGTCATGGCGATGCGG	Sequencing of *dprE1*
dprE1_3_Fwd	CAACGGTTTGACCGGCATCGTGG	Sequencing of *dprE1*
dprE1_3_Rev	TGCGTCGGGTGGGACCAGGAATTG	Sequencing of *dprE1*
dprE1_4_Fwd	AGCAGGTTTCGCCCAGCACCAATTC	Sequencing of *dprE1*
dprE1_4_Rev	GGTTGCCCGTGGCATCAATCATG	PCR amplification and sequencing of *dprE1* + ~200 bp downstream stop codon
sigA_1_Fwd	GAACATCAAGCAGACAGCCAAATTCTCG	PCR amplification and sequencing of *sigA* + ~200 bp upstream start codon
sigA_1_Rev	CTTCCTCTTCGGCGTTGAGCAG	Sequencing of *sigA*
sigA_2_Fwd	GAAAGACAAGGCTTCAGGCGATTTC	Sequencing of *sigA*
sigA_2_Rev	GTAGCAGCTCACGCTGGATACG	Sequencing of *sigA*
sigA_3_Fwd	ACAAGTTCTCGACGTACGCCACCTG	Sequencing of *sigA*
sigA_3_Rev	AATACGGCGAGCGTGAAGCCAG	PCR amplification and sequencing of *sigA* + ~200 bp downstream stop codon

[a] Primers were custom-synthesized by Genewiz Inc.

[b] PCR amplification of *M. abscessus dprE1* and *sigA* was carried out using the extracted genomic DNA of the mutant strains, Phusion DNA polymerase (Thermo Scientific) and primers “dprE1_1_Fwd” + “dprE1_4_Rev” (for *dprE1*) or “sigA_1_Fwd” + “sigA_3_Rev”. Sanger

sequencing of the amplified PCR product of *dprE1* was performed using primers "dprE1_1_Fwd", "dprE1_1_Rev", "dprE1_2_Fwd", "dprE1_2_Rev", "dprE1_3_Fwd", "dprE1_3_Rev", "dprE1_4_Fwd" and "dprE1_4_Rev". Sanger sequencing of the amplified PCR product of *sigA* was performed using primers "sigA_1_Fwd", "sigA_1_Rev", "sigA_2_Fwd", "sigA_2_Rev", "sigA_3_Fwd" and "sigA_3_Rev".
